# Kinetic and Thermal Study of Ethylene and Propylene Homo Polymerization Catalyzed by *ansa*-Zirconocene Activated with Alkylaluminum/Borate: Effects of Alkylaluminum on Polymerization Kinetics and Polymer Structure

**DOI:** 10.3390/polym13020268

**Published:** 2021-01-15

**Authors:** Amjad Ali, Nadeem Muhammad, Shahid Hussain, Muhammad Imran Jamil, Azim Uddin, Tariq Aziz, Muhammad Khurram Tufail, Yintian Guo, Tiantian Wei, Ghulam Rasool, Zhiqiang Fan, Li Guo

**Affiliations:** 1 Research School of Polymeric Materials, School of Material Science & Engineering, Jiangsu University, Zhenjiang 202113, China; amjadali@zju.edu.cn (A.A.); shahid@ujs.edu.cn (S.H.); 2211905055@stmail.ujs.edu.cn (T.W.); 2 Department of Enviromental Engineering, Wuhan University of Technology, Wuhan 430223, China; nadeem@zju.edu.cn (N.M.); Khurram.ch91@bit.edu.cn (M.K.T.); ghulam46@yahoo.com (G.R.); 3 MOE Key Laboratory of Macromolecular Synthesis and Functionalization, Department of Polymer Science and Engineering, Zhejiang University, Hangzhou 310027, China; jamil@zju.edu.cn (M.I.J.); auddin@zju.edu.cn (A.U.); Tariq_mehsud@yahoo.com (T.A.); guoyiantian@sinochem.com (Y.G.)

**Keywords:** metallocene, borate, ethylene, propylene, polymerization, kinetics, activator

## Abstract

The kinetics of ethylene and propylene polymerization catalyzed by homogeneous metallocene were investigated using 2-thiophenecarbonyl chloride followed by quenched-flow methods. The studied metallocene catalysts are: *rac*-Me_2_Si(2-Me-4-Ph-Ind)_2_ZrCl_2_ (Mt-I), *rac*-Et(Ind)_2_ZrCl_2_ (Mt-II) activated with ([Me_2_NPh][B(C_6_F_5_)_4_] (Borate-I), [Ph_3_C][B(C_6_F_5_)_4_] (Borate-II), and were co-catalyzed with different molar ratios of alkylaluminum such as triethylaluminium (TEA) and triisobutylaluminium (TIBA). The change in molecular weight, molecular weight distribution, microstructure and thermal properties of the synthesized polymer are discussed in detail. Interestingly, both Mt-I and Mt-II showed high activity in polyethylene with productivities between 3.17 × 10^6^ g/mol_Mt_·h to 5.06 × 10^6^ g/mol_Mt_·h, activities were very close to each other with 100% TIBA, but Mt-II/borate-II became more active when TEA was more than 50% in cocatalyst. Similarly, Polypropylene showed the highest activity of 11.07 10^6^ g /mol_Mt_·h with Mt-I/Borate-I/TIBA. The effects of alkylaluminum on PE molecular weight were much more complicated; MWD curve changed from mono-modal in Mt-I/borate-I/TIBA to bimodal type when TIBA was replaced by different amounts of TEA. In PE, the active center fractions [C*]/[Zr] of Mt-I/borate were higher than that of Mt-II/borate and average chain propagation rate constant (*k*_p_) value slightly decreased with the increase of TEA/TIBA ratio, but the Mt-II/borate systems showed higher *k*_p_ 1007 *k*_p_ (L/mol·s). In PP, the Mt-I/borate presented much higher [C*]/[Zr] and *k*_p_ value than the Mt-II. This work also extend to investigate the mechanistic features of zirconocenes catalyzed olefin polymerizations that addressed the largely unknown issues in zirconocenes in the distribution of the catalyst, between species involved in polymer chain growth and dormant state. In both metallocene systems, chain transfer with alkylaluminum is the dominant way of chain termination. To understand the mechanism of cocatalyst effects on PE M_w_ and (MWD), the unsaturated chain ends formed via *β*-H transfer have been investigated by ^1^H NMR analysis.

## 1. Introduction

On the way of synthesizing polyolefin with unique characterizations with narrow molar mass distributions and branching degree, academic and industrial researchers have devoted their consideration in polymer science and organometallic chemistry [1,2,3,4]. To design new polymerization technologies based on transition metal compound, homogeneous metallocene single-site catalyst precursors and main-group organometallic alkylaluminum cocatalysts can be considered valuable strategies to achieve this goal [5,6,7,8,9]. Homogeneous metallocene catalyzed polyolefin has become an interesting topic over the past decade and the commercialization of homogeneous metallocene catalyst is expected to be a revolution in the polymer industry [10,11,12,13]. However, the productivity of these polymerization catalysts has been significantly improved and produces polyolefin with narrow molecular weight distribution. In addition, soluble Ziegler–Natta polymerization catalysts based on zirconium are much more productive than heterogeneous Ziegler–Natta catalysts. Therefore, soluble Ziegler–Natta or homogeneous metallocene single-site catalyst can be used to synthesis new polymers without the need to remove the catalyst residues from the final polymer product. The activities of soluble catalysts are very encouraging for commercial purposes, but the kinetics behind these catalysts system have not been investigated extensively [14,15,16,17].

Kinetic method delivers not only an appreciated mechanistic understanding into catalyzed reactions but also has the potential to establish an extremely sensitive method to evaluate the effectiveness of the catalyst, cocatalyst [18,19,20]. Since the 1990s, the kinetic and mechanistic of the polyolefin have been widely studied and mechanistic investigation through detailed polyolefin chains structure characterization has made significant achievements [20,21,22,23,24,25] Historically, findings of novel and more effective and effective findings of kinetic models have contributed significantly to the technology as well as the fundamental understanding in this field. It is obvious that the kinetic studies based on direct counting of the [C*]/[Z] concentration in the polymer reaction system can permit the easier construction of a comprehensive mechanistic model. Many such studies have been reported based on different procedures of counting the active center concentration in homogenous metallocene catalyzed polymerization [26,27,28,29,30]. However, conflicting results of active centers values have been found in the literature while dealing with the same type of metallocene catalyzed polymerization, maybe because of different sensitivity levels of the methods that have been used [26,31,32].

With respect to the cocatalyst, there are indeed conceptions that describe the activation of homogeneous metallocenes catalyst through Alkylaluminum derivatives cocatalyst to form the active cation, alkylaluminum. Active cations like methylaluminoxane (MAO), triethylaluminum (TEA), and triisobutylaluminum (TIBA) are added as a scavenger of impurities and, more importantly, as alkylation agent, to form alkylated metallocene that is the precursor of cationic active centers [31,32,33]. However, the mechanism of alkylaluminum derivatives that affects the metallocene/borate catalyzed olefin polymerization and polymer structure has not been fully clarified. Till now, only five mechanistic studies are acknowledged about the role of alkylaluminum derivatives cocatalyst during the polymerization process. In principle, a suitable alkylaluminum cocatalyst is needed for conversion of transition metal such as Ti, Hf, Zr based metallocene complex into active species of olefins polymerization [34,35,36]. Alkylaluminum derivatives like methylaluminoxane (MAO) and modified methylaluminoxanes (MMAO) are a more efficient alkylaluminum cocatalyst because of their stronger Lewis acidity, weaker reduction ability and high steric bulk, but excess of the MAO is needed for activation of transition metal based metallocene complex. In contrast, using TEA, TIBA or TEA/TIBA mixture in combination with borate as activator can lead to the same level of catalytic activity as the metallocene/MAO system without the need of an excess amount of an expensive MAO. However, excessive use of alkylaluminum in the metallocene/borate/alkylaluminum system can reduce the metallocene’s catalytic activity. Therefore, an optimal quantity and type of alkylaluminum is required for olefin polymerization with borate cocatalyst. In the metallocene/MAO system, the MAO solution always contains “free” trimethylaluminum (TMA) that works as a reducing agent and chain transfer agent when it reacts with the metallocene complex and the active centers, and it can form Me-bridged dinuclear metallocene species which are thought to be catalytically inactive.

Our group has previously reported the effects of alkylaluminum cocatalyst (TEA, TIBA and TEA/TIBA mixtures) on ethylene, propylene polymerizations and E/P copolymerization with Z-N catalyst. Stronger alkylaluminum cocatalyst effects on the active center distribution and molecular weight and molecular weight distribution of polyolefin have been found, and the effects were explained by the different alkylation, reduction and chain transfer properties of different diverse alkylaluminus [37,38,39,40]. According to the best of our knowledge, the effects of cocatalysts on fundamental polymer reaction stage have not been studied so far, despite the homogeneous metallocene catalyst systems based on borate/alkylaluminum derivatives demonstrated the most attractive combination of selectivity, activity and generality for a wide-ranging variety of olefins. 

Hence, in this work, the effects of linear and branched alkylaluminum on polyethylene and polypropylene with zirconocenes/borate/alkylaluminum have systematically been investigated. By changing the alkylaluminum/zirconocene molar ratio and TEA/TIBA molar ratio in alkylaluminum component, Mw, MWD, microstructure and thermal properties of the obtained polymers are found to be strongly changed. It will be interesting to know how to clarify the mechanism of the observed effects, the changes of active center concentration as well as the apparent chain propagation constant with the alkylaluminum type and concentration. It will also be interesting to know how to determine the following effect by the quench-labeling method using 2-thiophenecarbonyl chloride (TPCC) as the quencher based on selectively quenching the metal-polymer bonds through acyl chloride, which has been verified in our previous study by application in the polymerization of olefins with Ziegler–Natta, nickel–diimine and metallocene catalysts [39,41,42,43,44]. Thus, it would be worthwhile to understand the mechanism of cocatalyst effects on PE molecular weight, MWD mono-modal to bimodal type and unsaturated chain ends formed via β-H transfer, which have investigated by ^1^H NMR and GPC (Flory components) analysis.

## 2. Experimental Section

### 2.1. Materials

All the reagents used were bought from commercial sources. The metallocene catalyst *rac*-Me_2_Si(2-Me-4-Ph-Ind)_2_ZrCl_2_ (Mt-I) was supplied by Shanghai Research Institute of Chemical Industry, Shanghai, China. *rac*-Ethylenebis(indenyl)zirconium dichloride (Mt-II) was purchased from Sigma-Aldrich (Hangzhou, China). Borates [Me_2_NPh] [B(C_6_F_5_)_4_] (Borate-I) and [Ph_3_C] [B(C_6_F_5_)_4_] (Borate-II) were kindly supplied by Sinochem Lantian, Zhejiang Research Institute of Chemical Industry Co., Ltd., Hangzhou China. The metallocenes and borates were respectively diluted in toluene and stored under nitrogen before use. Solutions of alkylaluminum such as TIBA (triisobutyl aluminum) and TEA (triethylaluminium) were made by the dissolving the required quantity of neat TIBA or TEA in *n*-heptane by the Schlenk line method.

All moisture and oxygen sensitive manipulations were carried out by using glove and Schlenk techniques. Both gases, i.e., ethylene and propylene (polymerization grade, 99.9% purity), were bought from Zhejiang mixing Gas Co. (Hangzhou, China). Further, they were purified by columns gas purification method containing 4 Å molecular sieves and deoxygenize agent in the purification system (Dalian Samat Chemicals Co., Ltd., Dalian, China). *N-Heptane* (Shanghai Titan Scientific Co., Ltd., Shanghai, China) was purified by passing through columns of deoxygenate agent and molecular sieve, refluxed over sodium-benzophenone and refined under nitrogen prior to use. Toluene solvent (HPLC grade, Jiangsu Yonghua Fine Chemical Co., Ltd., Changsha, China) was refluxed over sodium-benzophenone and distilled under the nitrogen before experiments. 

### 2.2. Polymerization

Polymerizations were conducted in a 100 mL glass reactor. In a standard experiment, the polymerizations reactor was heated at 95 °C for at least half hour under vacuum and then cooled down to the desired reaction temperature (50 °C). The glass reactor was flushed with nitrogen gas three times, and 50 mL of toluene solvent was charged, fallow by saturation with monomers gas of 0.1 MPa. The solution of alkylaluminum (TEA, TIBA or TEA/TIBA mixture) was added to the polymer reactor, respectively. Afterwards, the metallocene (dissolved in toluene) was introduced and the reaction run for 10 min to complete alkylation of the metallocene catalyst by alkylaluminum [21]. Lastly, the polymerization reactions was started by injecting the borate (dissolved in toluene) solution. During the polymerization process monomers gas of 0.1 MPa was continuously supplied to the reactor to compensate for the converted monomer. After the required time of polymerization, TPCC (solution in *n*-Heptane) at TIBA/TPCC or TEA/TPCC = 1/2 was introduced to quench the polymer reaction for 5 min; 5 mL of ethanol with 2–3 drop of HCl was also injected to totally decompose the unreacted quencher and the catalyst system. The obtained polymer was precipitated with an excess amount of ethanol. Finally, it was purified according to our previous work [26].

### 2.3. Characterization of Polymer

#### 2.3.1. Nuclear Magnetic Resonance Spectroscopy (^1^H-NMR and ^13^C-NMR)

Obtained polymers products were characterized by high temperature (120 °C) nuclear magnetic resonance spectroscopy (^1^H-NMR and ^13^C-NMR) using instrument Varian Mercury-300 spectrometer (Hangzhou, China) operating 75 MHz within acquisition time 3.0 s. To obtai ^13^C-NMR spectra for quantitative determination, the estimated amount (2–3 mg) of relaxation agent Cr(acac)_3_ was added to the sample tube, and 1,1,2,2-tetrachloroethane-d_2_ was used as the solvent for NMR analysis at 120 °C [45,46,47].

#### 2.3.2. Gel Permeation Chromatography (GPC)

The molecular weight (M_w_) and molecular weight distribution (MWD) of produced polymers were determined by using a PL-220 gel permeation chromatography (GPC, Polymer Laboratories, Shrophire, Uk) With refractive index detector (RID, Polymer Laboratories, Shrophire, UK) at 150 °C using 1,2,4-trichlorobenzene as eluent. The device was equipped with three PL-MIXED-B (300 mm × 7.5 mm) columns, as well as a 50 mm guard column. The polymer samples were dissolved in 1,2,4-trichlorobenzene at 135 to 140 °C with the concentration of 0.3–0.4 wt %. The eluent flow rate was set at 1 mL/min. The columns were calibrated with narrow molar mass distribution polystyrene standard samples. Molecular weight was calculated by the method of universal calibration method. 

#### 2.3.3. Differential Scanning Calorimetry (DSC)

Differential scanning calorimetry (DSC, Hangzhou, China) characterization was done with a TA Q200 instrument calibrated with indium and water. Four to six mg of each polymer was weighted and sealed into the sample pan. The thermal analysis of the polymers samples were conducted under the nitrogen atmosphere. The polymer samples was first heated from room temperature to 150 °C (for PE) or 180 °C (for PP) at 10 °C/min, and kept for 2–5 min, then cooled to −60 °C at 10 °C/min, and kept for 2–5 min. The polymer sample was heated again to 150 °C (for PE) or 180 °C (for PP) at 10 °C/min. the heat flow of the second heating scan was recorded as the thermal curve for each sample.

#### 2.3.4. Sulfur Content of the Quench-Labeled Samples

To count the active sites, the sulfur content of the quenched and purified polymer samples was measured in an YHTS-2000 ultraviolet fluorescence sulfur analyzer with a lower detection limit of 0.05 ppm (Jiangyan Yinhe Instrument Co., Jiangyan, China). The polymer samples for analysis were in the form of rubbery or powder materials for further analysis. 3 to 5 mg of the samples was weighed for each analysis run of with four parallel measurements, and the average value was taken as the sulfur content. The sulfur content of a blank polyethylene sample synthesized at the same conditions without the quenching step was found to be nearly 0, in contrast to the 5–25 ppm sulfur content of the quench-labeled samples. The relative error of the measurement was about 5% [26,30].

## 3. Results and Discussion

### 3.1. Ethylene Polymerization: Activity and Polymer Structure

Ethylene polymerization at 0.1 MPa monomer pressure was conducted with Mt-Mt-II and I activated by different borate/alkylaluminum combinations (Borate-I, Borate-II, TEA and TIBA), respectively. The alkylaluminum/Zr molar ratio was fixed at 800. The other reaction parameters are described in Table 1 and Table 2. It is obvious that both Mt-I and Mt-II showed high activity in ethylene polymerization, with productivities between 3.17 × 10^6^ g/mol_Mt_·h to 5.06 × 10^6^ g/mol_Mt_·h. In our previous study on ethylene polymerization with Mt-II/MMAO and Mt-II/dMAO, productivities of 2.16 × 10^6^ g/mol_Mt_·h and 5.2 × 10^6^ g/mol_Mt_·h were observed, respectively [30]. The borate/alkylaluminum cocatalyst showed similar activity as that of aluminoxane-based cocatalyst.

**Figure 1:**
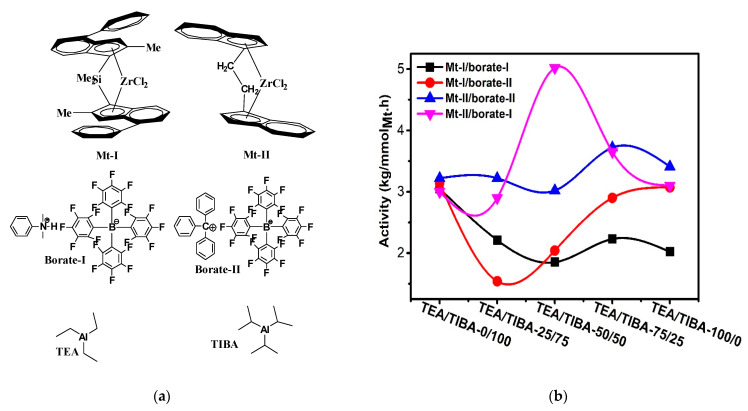
(**a**) Metallocene catalysts, borates and alkyl aluminums used in this work; (**b**) changes of activity with type of alkylaluminum triisobutylaluminium (TIBA)/triethylaluminium (TEA) mixtures.

As seen in Table 1 and Table 2, gradually changing the alkylaluminum from pure TIBA to TEA/TIBA mixture and finally pure TEA caused moderate variation of catalytic activity. In the cases of Mt-II/borate-II systems, higher activity is observed with TEA/TIBA mixture of 50/50 molar ratio (run 2.8 in Table 2, but further increase of TEA amount caused a decrease in activity. In contrast, adding TEA in Mt-I/borate systems caused a decrease in activity, though the extent of the activity decrease was not so large (see Figure 1b). Normally, the addition of TIBA in metallocene/MAO system led to an increase in the polymer’s molecular weight, while adding TEA or TMA caused a decrease in molecular weight [48]. However, in metallocene/borate systems, the effects of alkylaluminum on PE molecular weight were much more complicated. For the Mt-II/borate I systems, increasing the TEA/TIBA ratio in the alkylaluminum from 0 to 50/50 has caused an evident increase of the M_w_; meanwhile, the MWD was moderately broadened. The further increase of the TEA/TIBA mole ratio decreased the M_w_ and slightly narrowed MWD. As compared with the Mt-II/MMAO system that produced PE with M_w_ of higher than 100,000 under similar conditions [20], the very low M_w_ (<5000) of PE produced with Mt-II/borate I/TIBA or Mt-II/borate I/TEA is quite strange.

**Figure 2:**
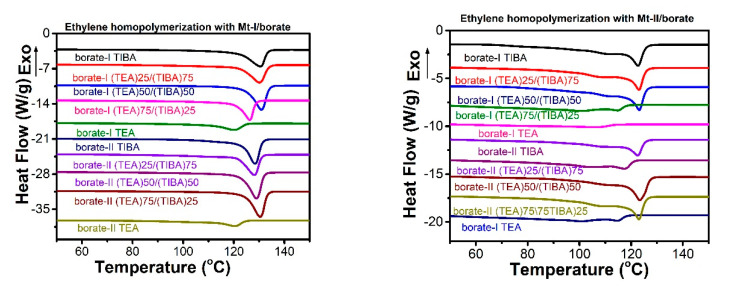
Differential scanning calorimetry (DSC) heating traces of PE produced with both metallocenes under TEA/TIBA mixture of different ratios.

The lack of high molecular weight polymer chains in PE produced with Mt-II/borate-I means that chain transfer with alkylaluminum was very fast in this system, and the structure of alkyls in the alkylaluminum only slightly influence the efficiency of chain transfer with alkylaluminum. This phenomenon will be further discussed in a later part of this section. In contrast to the Mt-II/borate-I/alkylaluminum systems, the Mt-I/borate-I/alkylaluminum catalysts produced PE with much higher M_w_ and significantly broader MWD. When pure TIBA was the alkylaluminum, the M_w_ (163,000) was very close to that of the PE produced with Mt-II/MMAO (195,000) reported in the literature [49], but the polydispersity of PE by Mt-I/borate I/TIBA (Ɖ = 10.3) was more than three times larger than that of Mt-II/MMAO (Ɖ = 3.05). Similarly, when TEA was introduced in the Mt-I/borate I catalyst, the PE molecular weight sharply decreased with increase of the TEA/TIBA ratio, and the polydispersity index first increased then decreased (see Table 1). As shown in Figure 2, the MWD curve changed from mono-modal in Mt-I/borate I/TIBA to a bimodal type when TIBA was replaced by a different amount of TEA. This kind of complicated MWD change by alkylaluminum has not been reported in the literatures before. 

The melting temperature and melting enthalpy of PE with both metallocenes have been determined by DSC thermal analysis, as shown in Figure 2, and the data of thermal properties have been listed in Table 1 and Table 2. 

PE synthesized with Mt-I/borate showed a melting temperature of 120–30 °C, and melting of 106–225 J/g, and indicates more consistency in melting temperature; while Mt-II/borate produces a PE melting temperature of 101–123 °C and melting enthalpy of 156–218 J/g, With an increase of TEA content in the alkylaluminum cocatalyst, the melting enthalpy increased evidently, but pure TEA in cocatalyst shows a decrease in melting temperature and melting enthalpy. The melting temperature of PE is mainly associated with the short chain branching density. The higher the short chain branch density, there is a decline in lamellar thickness of the crystal structure, and this therefore shows the lower melting temperature of the polyethylene.

### 3.2. Ethylene Polymerization: Chain Transfer Reactions

**Scheme 1:**
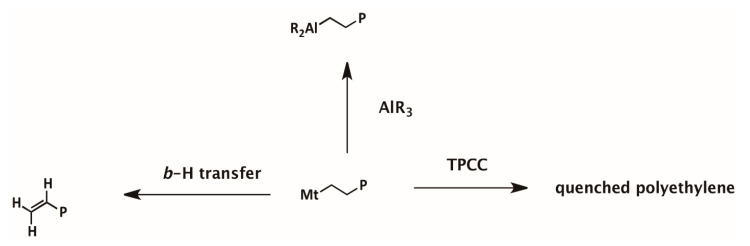
Mechanism of chain transfer reactions in ethylene polymerization with metallocene /borate/alkyl aluminum.

**Figure 3:**
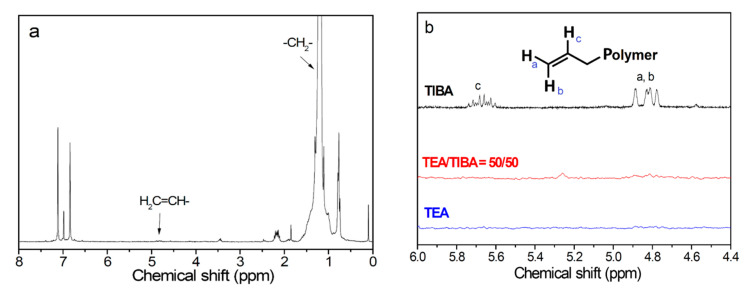
^1^H NMR spectra of polyethylene ((see supporting information) (**a**). Run 1.1, 1.3, 1.5 in Table S1, (**b**). Runs 2.1, 2.3 and 2.5 in Table S1).

To understand the mechanism of cocatalyst effects on PE molecular weight and MWD, the unsaturated chain ends formed via *β*-H transfer have been investigated by ^1^H NMR analysis on the polymer. According to the chain transfer mechanism, as shown in Scheme 1, *β*-H transfer of a propagating chain forms a vinyl ended polyethylene chain. Chain transfer with alkylaluminum will lead to PE chains with saturated chain ends. In the ^1^H NMR spectra of PE samples in Table 1 and Table 2, the multiple peaks at 4.8–5.1 ppm and 5.6–5.8 ppm observable in some samples were assigned to protons of the vinyl end group (see Figure 3).

Other kinds of double bond (vinylidene and vinylene) cannot be detected or showed very weak signals in the ^1^H NMR spectra, so they are neglected in counting the unsaturated end groups. The total chain number of synthesized PE (*N*_pol_) was calculated by the following equation:

(1) $$N_{\mathrm{pol}}=\frac{m_{\mathrm{pol}}}{\bar{M_{n}}}$$

where *m*_pol_ is the mass of the PE sample, and *M*_n_ is the number average molecular weight determined by GPC analysis. Since the vinyl group is the only unsaturated end group present in PE, the number of saturated end groups (*N*_Al_) can be calculated by *N*_Al_ = *N*_pol_ − *N*_v_ (*N*_v_ is the number of the vinyl end group determined by ^1^H NMR analysis). 

The number of PE chains formed via different chain transfer reactions are summarized (see supporting information) in Table SI. It could be found that chain transfer with alkylaluminum is the only way of chain termination in most PE samples. In ethylene polymerization with Mt-II/borate/TIBA, vinyl ended PE accounted for about 70% of the chains, while in the Mt-I/borate/TEA system, vinyl ended PE accounted for about 10%. It means that the Mt-I/borate/alkylaluminum system produced PE with dominantly saturated end groups regardless of the type of alkylaluminum.

In this system, the M_w_ of PE evidently decreased with an increase of TEA in the cocatalyst, meaning that the chain transfer with the Al-Et bond should be faster than that with the Al-*i*Bu bond. Larger steric bulkiness in Al-*i*Bu than Al-Et could be responsible for their different chain transfer efficiencies. In the case of Mt-II/borate/alkylaluminum systems, chain transfer via *β*-H transfer became the major way of chain termination only when TIBA was used as cocatalyst, and most of the PE chain ends were saturated when TEA was added. Since the rate of chain transfer with alkylaluminum is not be influenced by monomer concentration, it can be expected that molecular weight of PE has been increased by raising ethylene pressure in ethylene polymerization with Mt-I/borate/alkylaluminum and Mt-II/borate/TEA.

### 3.3. Ethylene Polymerization: Active Center Fraction and Mechanism

The method of counting active center concentration by the quench-labeling method using TPCC as the quencher has been successfully applied in this study on ethylene and propylene polymerization with Mt-I/borate/TIBA and Mt-II/borate/TIBA. This method has been used in our previous systematical comparative studies on the kinetics of ethylene and propylene polymerizations with Mt-I and Mt-II using borate/alkylaluminum as the activator [30]; the same method has been used to study the effects of alkylaluminum cocatalyst on active fraction ([C*]/[Zr]). Detailed kinetics of zirconium catalyzed ethylene polymerization provides key information for understanding the mechanism of these important industrial processes. As indicated by Busico et al. [50,51], the chain propagation constant (*k*_p_) can be calculated using the “deceptively simple” rate law (Equations (2) and (3)) as follows:

*R*_p_ = *k*[M] (2)

**Figure 4:**
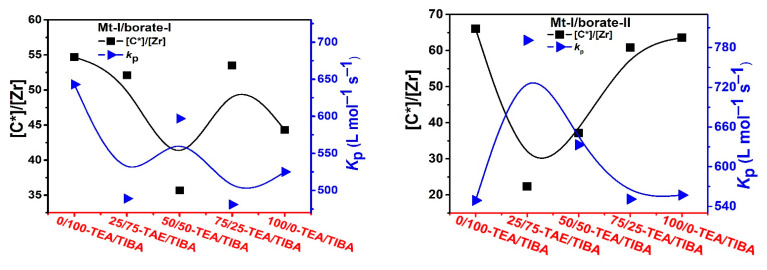
Active center ratio and *k*_P_ of ethylene polymerization with Mt-I/borate/alkylaluminum.

**Figure 5:**
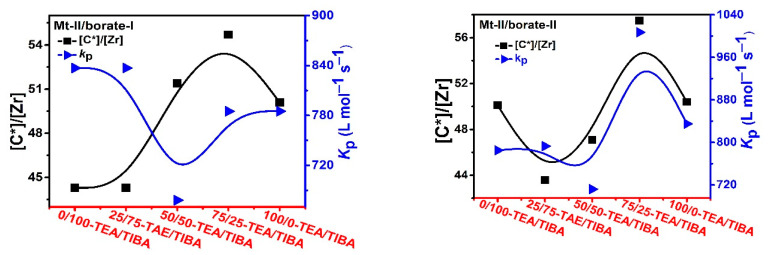
Active center ratio and *k*_P_ of ethylene polymerization with Mt-II/borate/alkylaluminum.

*R*_p_ = *k*p [C*][M] (3)

where [M] is monomer equilibrium concentration, [E] = 0.085 mol/L in toluene at 50 °C and 0.1 MPa was used for the calculation [26,52]. Since the instant polymerization rate at the time of TPCC quenching (*t*_p_ = 20 min) was not determined, the average *R*_p_ value calculated from the polymer yield after 20 min polymerization and the catalyst amount has been used. The *k*_p_ value thus obtained is an average value in the 20 min polymerization process. When we compare the average *k*_p_ value of different polymerization runs conducted under the same conditions for the same period, it is possible to draw meaningful information on the alkylaluminum effects on the intrinsic reactivity of the active centers. Figure 4 and Figure 5 show the active center fraction and average propagation rate constant of Mt-I/borate/alkylaluminum and Mt-II/borate/alkylaluminum systems, respectively. The detailed data have been shown in Table 1 and Table 2.

As shown in Figure 4, the active center fractions of Mt-I/borate/alkylaluminum catalyzed ethylene polymerization were higher than 50%, when TIBA was present in the cocatalyst (the lower active center fractions of the systems with TEA/TIBA mixtures could be attributed to experimental error), and the system with only TEA showed a slightly lower [C*]/[Zr] fraction, meaning that activation of Mt-I by the borate/alkylaluminum cocatalyst was not evidently influenced by the type of alkylaluminum. 

Since the molecular weight of PE was strongly depressed by increasing the amount of TEA, and chain transfer with alkylaluminum was the major way of chain termination irrespective of the type of alkylaluminum, we can conclude that chain transfer with an Al–Et bond is more efficient than that with an Al–*i*Bu bond. In other words, it is feasible to regulate PE molecular weight by adding TEA in the catalytic system. By comparing the *k*_p_ values of Mt-I/borate/alkylaluminum systems with different TEA/TIBA ratios, we can see that the effects of the alkylaluminum type were rather weak. In Mt-I/borate-I/alkylaluminum systems, the *k*_p_ value slightly decreased with the increase of the TEA/TIBA ratio. When borate-II was used as activator, the change of *k*_p_ value was negligible. These results seem to imply that the active centers formed in the presence of different alkylaluminum cocatalysts are similar to each other.

In the case of ethylene polymerization with Mt-II/borate in the presence of TEA/TIBA, similarly weak effects of alkylaluminum type on active center fraction and *k*_p_ value can be observed (see Figure 5). In comparison with the Mt-I/borate/alkylaluminum catalysts, the Mt-II/borate/alkylaluminum systems gave a lower [C*]/[Zr] fraction (44–55%), but larger *k*_p_ value (700–850 L/mol.s). The stronger steric hindrance in the molecular structure of Mt-I than that of Mt-II could be responsible for their different *k*_p_ values, since lower steric hindrance in the metallocene active sites allows for more efficient olefin coordination and insertion. As to the higher [C*]/[Zr] fraction in Mt-I, the electronic effects of its substituents on indenyl ligand (methyl at position 2 and phenyl at 4) could be the main reason, since electron donating substituents have been found to be beneficial to abstracting anions from neutral metallocene complexes.

**Figure 6:**
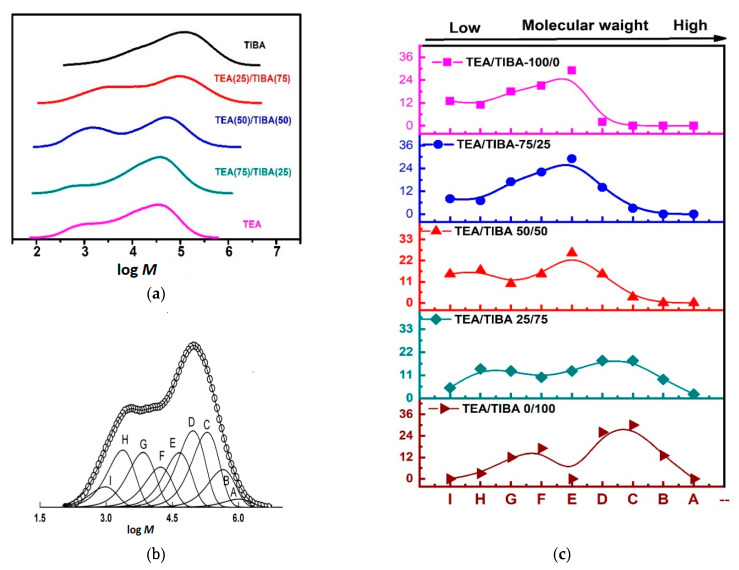
(**a**) MWD curves of PE produce with Mt-I/borate-I and TEA/TIBA mixtures, (**b**) deconvolution of PE MWD curve into Flory components (run 1.2 in Table 1, (**c**) distribution of Flory components of PE produced from Mt-I/Borate-I with various mixtures of TIBA/TEA.

Figure 6a shows that MWD curves of PE produced with Mt-I/Borate-I/alkylaluminum became bimodal when TEA was introduced in the catalytic system. To explain the reasons for this phenomenon, each MWD curve was deconvoluted into six to nine Flory “most probable” distribution functions (Flory components) with satisfactory accuracy according to the method reported in the literature [37,53]. A typical MWD curve and the deconvoluted Flory components are shown in Figure 6b. 

The Flory components are named **A**, **B** … to **I** in the order of descending molecular weight. Supporting information Table S2, shows the fraction (relative intensity) and weight average molecular weight of the deconvoluted Flory components. It was found that six Flory components could be observed in polyethylene produced using either TIBA or TEA as coactivator, while more components were seen using a TIBA/TEA mixture as coactivator. As proposed in the previous work of our group, polyethylene chains with high molecular weight are formed by active centers composed of contact ion pairs, which have a fast rate of chain propagation but a much slower rate of chain transfer reaction due to the steric hindrance of the counter ion. 

On the contrary, active centers with loosely associated ion pairs would form low molecular weight polymer, since this type of active centers has both fast chain propagation and fast chain transfer reaction [37]. By comparing the distribution of Flory components in different PE samples (see the supporting information), it will be possible to understand the effects of alkylaluminum cocatalyst on the active center distribution, and make further discussions on the related mechanism.

Figure 6c schematically shows the distribution of Flory components in PE samples and the weight fraction of the Flory component (see supporting information, Table S2). It is seen that components B and C only existed with high content (>9%) in PE produced with pure TIBA or 25/75 TEA/TIBA mixture, while components H and I only existed with high content (>6%) in PE produced with TEA as the dominant alkylaluminum (see supporting information, Figures S1–S5).

The general trend of alkylaluminum effects on the Flory component distribution can be summarized as follows: with the rise of TEA content in cocatalyst from 0 to 100%, at first the Flory components with low molecular weight (components F, G, H and I) were enhanced; meanwhile, those with high molecular weight became weaker. For example, total fraction of components F–I was increased from 32% in PE formed with 100% TIBA to 42% in PE formed with 25/75 TEA/TIBA mixture. A further rise of TEA content in the cocatalyst from 25% to 50% and finally to 100% caused an evident shift of the high molecular weight components to the low molecular weight side (compare Figure 3). It means that the components forming a low molecular weight PE (**F**–**H**) are more sensitive to the Al–Et bond in the cocatalyst, and enhancement of chain transfer with alkylaluminum in the components forming high molecular weight PE (**A**–**D**) requires a higher percentage of Al–Et bond in the cocatalyst. The different efficiency of chain transfer with Al–Et and Al–*i*Bu bonds could be explained by their different steric bulkiness. With larger steric hindrance of the isobutyl in Al–*i*Bu, the surrounding of active centers became more sterically crowded, and the rate of chain transfer reaction became slower, leading to polyethylene with higher molecular weight. With smaller alkyl-like ethyl in Al–Et, the smaller steric hindrance would be beneficial for fast chain transfer with the Al–Et bond.

In our earlier study on ethylene polymerization with Mt-II/MMAO, three kinds of active centers were discriminated, in which the active centers with the highest *k*_p_ and a loose ion pair produce polymers with the lowest molecular weight, and the active centers with lower *k*_p_ and a tight ion pair produce polymers with the highest molecular weight [26]. According to the literature, the TEA/TIBA mixture undergoes quick alkyl exchanges to form AlEt_2_(*i*Bu) and AlEt(*i*Bu)_2_ [54]. Thus, the TEA/TIBA mixture is a mixture of AlEt_3_, AlEt_2_(*i*Bu), AlEt(*i*Bu)_2_ and Al*i*Bu_3_, and the proportion of Al–Et bond will increase with the increase of TEA/TIBA molar ratio. At a TEA/TIBA molar ratio lower than 50/50, the content of AlEt_2_(*i*Bu) will be lower than AlEt(*i*Bu)_2_. It seems that chain transfer with AlEt(*i*Bu)_2_ in the active centers with tight ion pair is not much different from chain transfer with TIBA, possibly for the strong steric hindrance of AlEt(*i*Bu)_2_. Meanwhile, chain transfer with AlEt(*i*Bu)_2_ in the active centers with a loose ion pair (producing low molecular weight PE) can take place efficiently, since they have a larger space to adapt the bulky AlEt(*i*Bu)_2_. When there is a large amount of AlEt_2_(*i*Bu) in alkylaluminum mixtures of high TEA/TIBA ratio, efficient chain transfers with AlEt_2_(*i*Bu) can take place in the active centers with a tight ion pair, since AlEt_2_(*i*Bu) is less bulky than AlEt(*i*Bu)_2_. By these discussions, the complicated changes of PE MWD with cocatalyst structure can be largely understood.

### 3.4. Propylene Polymerization: Active Center Fraction and Mechanism

**Figure 7:**
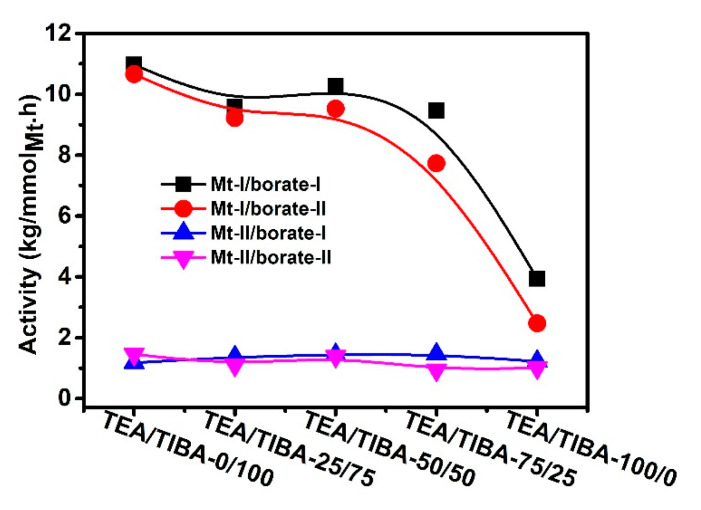
Effects of alkylaluminum on activity of propylene polymerization.

In this section, the effects of alkylaluminum cocatalyst on propylene polymerization with Mt-I and Mt-II activated by different borate/alkylaluminum combinations were investigated. The polymerization results, including active center fraction and average propagation rate constant, are listed in Table 3 and Table 4. As seen in Figure 7, the activity of Mt-I gradually decreased with an increase of the amount of TEA in the cocatalyst, but the activity loss was only slight when there was a small amount of TIBA in the system. The activity was reduced to about 30% of the system with pure TIBA when only TEA was added. It means that TIBA is a key component of the cocatalyst for Mt-I to show high activity in propylene polymerization. The type of cation in the borate (PhN^+^HMe_2_ in borate-I and Ph_3_C^+^ in borate-II) had only slight influence on the activity. The molecular weight of PP produced with Mt-I/borate-I tended to decrease with an increase of TEA in the cocatalyst. Unlike the very broad and sometimes bimodal MWD of PE produced with the same catalytic system, propylene polymerization gave PP with relatively narrow MWD, with the polydispersity index not higher than 4.5. It is interesting to see that the polydispersity index of PP decreased with the increase of TEA in the cocatalyst. The related mechanism will be discussed later in this paper.

In contrast to Mt-II, the effects of alkylaluminum on activity of Mt-II/borate showed a different trend. The activity was only slightly changed with an increase of TEA content. In comparison with the activity of propylene polymerization with Mt-II/MMAO (3.8 × 10^6^ g/mol_Mt_·h) under the same conditions [20], the Mt-II/borate/alkylaluminum system showed evidently lower activity. 

However, the molecular weight of PP produced with Mt-II/borate-I/TIBA (60 kg/mol) was much higher than that of the Mt-II/MMAO system (15 kg/mol). Similar to the Mt-I/borate-I systems, the molecular weight of PP produced with Mt-II/borate-I also decreased with an increase of TEA in the cocatalyst. The two zirconocenes Mt-I and Mt-II studied in this study showed markedly different activity in propylene polymerization, with Mt-I (the highest activity was 11 × 10^6^ g/mol_Mt_·h) being about seven times more active than Mt-II (the highest activity was 1.4 × 10^6^ g/mol_Mt_·h). Similarly, the same difference between Mt-I and Mt-II has been reported by Spaleck et al., who used MAO as the cocatalyst [55]. The changes of polypropylene chain structure with a different cocatalyst may disclose important evidence of the catalytic process. The microstructure of some PP samples in Table 3 and Table 4 was analyzed with ^13^C NMR spectroscopy using peak assignments reported in the literature [56,57,58].

In Mt-I/borate-I systems, changing the alkylaluminum from TIBA to TEA caused a slight decrease of isotacticity ([*mmmm*]) from 76.9% to 75.2%. Similarly, in Mt-I/borate-II systems [*mmmm*] of PP was 89.3% in the presence of TEA and 90.1% in the presence of TIBA. [*mmmm*] of PP formed with TEA/TIBA mixture fell in between 87–91%. On the other hand, [*mmmm*] of PP formed with Mt-II/Borate-I/TEA was 62.9%, and that with Mt-II/Borate-II/TEA was 60%. Both metallocenes produced PP with higher isotacticity ([*mmmm*]) by using borate-II rather than borate-I as the activator, and the effect of alkylaluminum structure on PP isotacticity was rather weak. The lower stereoselectivity of Mt-II than Mt-I has also been reported in the literature [55]. 

**Figure 8:**
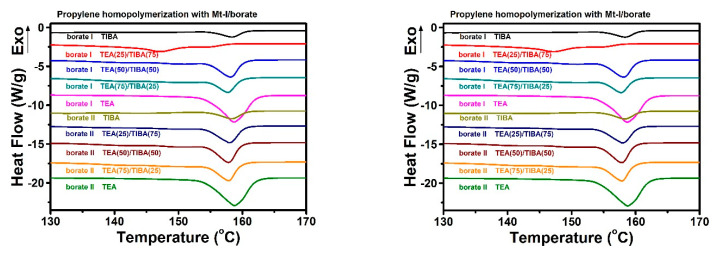
DSC heating traces of PP produced with Mt/borate under TEA/TIBA mixtures of different ratios.

The melting temperature and melting enthalpy of PP samples were determined by DSC thermal analysis. The DSC curves are shown in Figure 8, and the data of thermal properties are listed in Table 3 and Table 4. PP synthesized with Mt-I/borate showed melting temperature of 157–59 °C, and melting enthalpy of 70–120 J/g, which are similar to PP produced by supported Ziegler–Natta catalysts. With an increase of TEA content in the alkylaluminum cocatalyst, the melting enthalpy increased evidently. On other side, Mt-II produced PP with more stereo- and regiochemical errors, leading to markedly lower melting temperature (90–122 °C) and lower melting enthalpy (about 50% of the PP produced with Mt-I).

**Figure 9:**
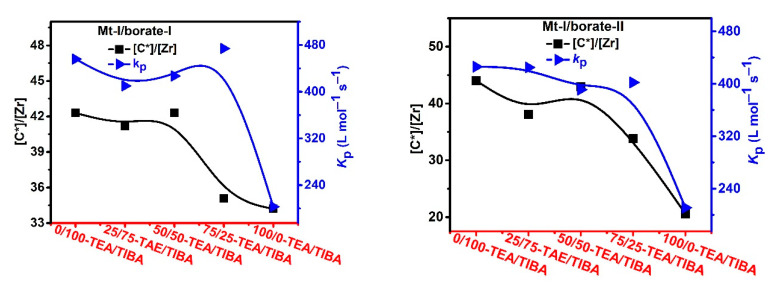
Active center ratio and *k*_P_ of propylene polymerization with Mt-I/borate/alkylaluminum.

**Figure 10:**
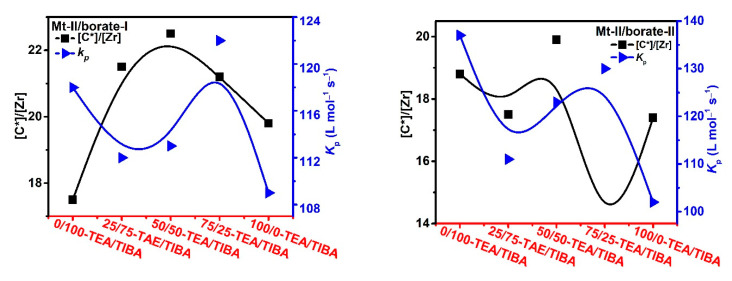
Active center ratio and *k*_P_ of propylene polymerization with Mt-II/borate/alkylaluminum.

To collect more evidence for explaining the observed cocatalyst effects in metallocene catalyzed propylene polymerization, active center fraction ([C*]/[M]) of the polymerization runs in Table 3 and Table 4 were determined by the TPCC quench-labeling method, and the results are shown in Figure 9 and Figure 10. It can be found that the active center fraction in propylene polymerization was evidently lower than that in ethylene polymerization. The active center fraction of Mt-I/borate/alkylaluminum was not higher than 44% in propylene polymerization, but was higher than 60% in ethylene polymerization. In the case of Mt-II/borate/alkylaluminum, the [C*]/[M] level was lower than 26.0% in propylene polymerization, in contrast to Mt-I, 45–57% in ethylene polymerization. 

Similar phenomena have been found in the previous studies of our group on ethylene and propylene polymerizations with Mt-II/MMAO, where the [C^*^]/[M] level was about 70% in the ethylene system and lower than 30% in propylene polymerization (see Table 5) [30,39,51] It has been explained that the active centers producing polypropylene should be loosely associated ion pairs but not contact ion pairs, since the high steric hindrance in active centers with contact ion pairs will prohibit coordination of the bulky propylene, but allow for ethylene coordination. Therefore, the lower [C*]/[M] level in propylene polymerization can be largely attributed to absence of contact ion pairs in its active center family.

In Figure 9 Mt-I/borate systems, increasing TEA in the cocatalyst caused decrease in [C*]/[M], especially when borate-II was used as the activator. [C*]/[M] of Mt-I/borate-II/TEA system was only about half of the Mt-I/borate-II/TIBA system. In the case of Mt-II/borate, an increase of TEA in the cocatalyst caused a slight increase in [C*]/[M], but further increase of the TEA/TIBA ratio caused a decrease in [C*]/[M]. In comparison with the Mt-I/borate systems, the alkylaluminum structure exerted rather weak influence on [C*]/[M] of Mt-II/borate systems (see Figure 10).

The effects of the alkylaluminum structure on *k*_p_ value were stronger in the Mt-I/borate systems. The rather high *k*_p_ value of Mt-I/borate/TIBA and Mt-I/borate/TIBA/TEA systems was reduced for about 50–60% when pure TEA was used as the cocatalyst, meaning that TIBA is a necessary component for high *k*_p_ of these systems. On the other side, effects of alkylaluminum structure on *k*_p_ value of Mt-II/borate systems were only moderate. The *k*_p_ value was reduced for about 20% when TIBA was replaced by TEA.

It is worth noting that both [C*]/[M] and *k*_p_ values of Mt-II/borate systems were evidently lower than that of Mt-I/borate systems in propylene polymerization. It means that the markedly lower activity of Mt-II than Mt-I is caused by lower efficiency of metallocene activation and slower monomer coordination in the former. The different stereochemical and electronic effects in these zirconocenes should be responsible for their different kinetics characteristics.

## 4. Further Discussion

Generally, activation of metallocene (L_2_M_T_X_2_ or L_2_M_T_R_2_) includes abstraction of an X^-^ or R^-^ anion from metallocene by MAO or borate, which leads to formation of cationic active center (L_2_M_T_R^+^). When borate is used as the activator for L_2_M_T_X_2_ type metallocenes, alkylaluminum like TIBA must be added to scavenge the impurities and alkylate the metallocene. In such a catalytic system, the structure and nature of both borate and alkylaluminum cocatalyst can obviously affect the catalyst activity, polymerization kinetics and the polymer’s chain structure. In this study, by combining investigations based on active center counting and polymer structure characterization, we have collected plenty of information that enables us to make further discussions on the mechanism of alkylaluminum effects in ethylene and propylene polymerization with zirconocene/borate. 

In ethylene polymerization with Mt-I/borate/alkylaluminum, the MWD of PE was broadened and the two GPC peaks in bimodal MWD became more separated when TEA content in alkylaluminum was increased. Meanwhile, molecular weight of PE markedly decreased with an increase of the TEA/TIBA ratio. This phenomenon can be explained by a more efficient chain transfer of the active centers with the Al–Et bond than with the Al–*i*Bu bond. The slight decrease of the chain propagation rate constant with the increase of the TEA/TIBA ratio could be attributed to an increase in the proportion of dormant active sites that cannot be coordinated by ethylene for too short a distance between the metallocenium cation and counter anion. According to the literature [1,26,53], [L_2_M_T_R] ^+^[B(C_6_F_5_)_4_]^−^ type ion pair has been proposed as a model of an active center in borate activated metallocene catalysts. 

In this model, initiation of polymerization requires dissociation of the ion pair to leave space for approaching and coordination of the olefin to the transition metal. When TEA is used as the cocatalyst, alkylation of the zirconocene forms L_2_M_T_(C_2_H_5_)_2_, which then forms the primary active center [L_2_M_T_(C_2_H_5_)]^+^[B(C_6_F_5_)_4_]^−^ after activation by borate. Because of the smaller size of Et than that of *i*Bu, the cation–anion distance in [L_2_M_T_(C_2_H_5_)]^+^[B(C_6_F_5_)_4_]^−^ should be shorter than that in [L_2_M_T_(*i*-C_4_H_9_)]^+^[B(C_6_F_5_)_4_]^−^ of the metallocene/borate/TIBA system, making its initiation (insertion of the first monomer in M_T_-R bond) more difficult. This factor may be the reason for the lower [C*]/[M] value of Mt-I/borate/TEA system than that of the Mt-I/borate/TIBA system in ethylene polymerization. 

The same mechanism can be applied to explain the much lower [C*]/[M] value of the Mt-I/borate/TEA system than that of the Mt-I/borate/TIBA system in propylene polymerization. Since propylene is a bulkier monomer than ethylene, the initiation efficiency of [L_2_M_T_(*i*-C_4_H_9_)]^+^[B(C_6_F_5_)_4_]^−^ will be markedly higher than that of [L_2_M_T_(C_2_H_5_)]^+^[B(C_6_F_5_)_4_]^−^ in the former system. It is thus understandable that the difference of [C*]/[M] between TEA and TIBA systems is larger in propylene polymerization.

**Scheme 2:**
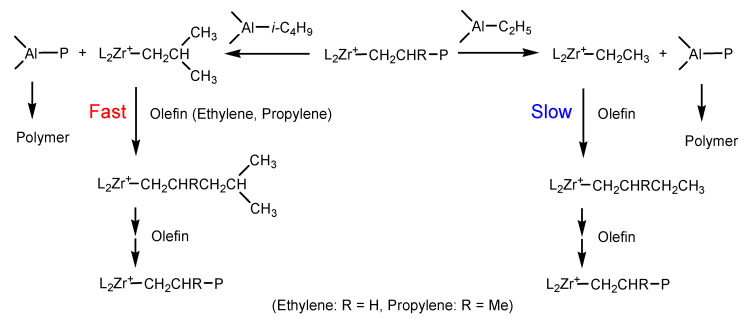
Chain transfer reactions of metallocenium active center with different Al-alkyl bonds of alkylaluminum.

The effects of alkylaluminum on *k_p_* values of ethylene and propylene polymerization can also be discussed based on the same active center model. Significant reduction of *k_p_* value by increasing TEA content was only observed in propylene polymerization catalyzed by Mt-I/borate. This phenomenon could be explained by a faster chain with Al–Et bond introduced by TEA, and slower initiation of [L_2_M_T_(C_2_H_5_)]^+^[B(C_6_F_5_)_4_]^−^ species formed in the chain transfer. When chain transfer with the Al–*i*Bu bond takes place in the same catalytic system, initiation of [L_2_M_T_(*i*-C_4_H_9_)]^+^[B(C_6_F_5_)_4_]^−^ will be much faster, and chain propagation will resume more quickly, leading to a high *k_p_* value (see Scheme 2). Molecular weights of PE and PP synthesized by Mt-I/borate have been found to be markedly reduced by adding TEA, which is caused by faster chain transfer with the Al–Et bond. In the case of Mt-II/borate, reduction of *k_p_* by increasing TEA content was rather weak, since reduction of molecular weight by adding TEA in the system was only moderate. In addition, the different kinetic behaviors of Mt-I and Mt-II systems can be tentatively explained by shorter cation–anion distance (active centers composed of tight ion pairs) than those with a longer cation–anion distance (loose ion pairs). As presented in Table 1 and Table 2, the (k_P_) value of the Mt-I catalyzed ethylene polymerization was lower than that of the Mt-II; meanwhile, it produced PE with higher M_w_. It means that [C*]/[M] in the Mt-I has shorter cation–anion distances than the Mt-II. Presence of methyl and phenyl substituents in Mt-I could be responsible for its tighter ion pairs.

## 5. Conclusions

In this study, the kinetics and mechanism **of** ethylene, propylene homopolymerizations with *ansa*-zirconocenes activated by alkylaluminum/borate cocatalysts, were systematically studied. The kinetic rate constant of chain propagation (*k*_p_) was determined based on the data of polymerization rate and active center fraction. By changing the TEA/TIBA molar ratio (0/100, 25/75, 50/50, 75/25 and 100/0) in the alkylaluminum component, polymerization activity as well as polymer’s M_W_, MWD, microstructure and thermal properties were found to be strongly changed. Activity of PE with Mt-I/borate was reduced by adding TEA in the cocatalyst, but the system with 100% TEA showed only slightly lower activity than the system with 100% TIBA. In PE with Mt-II/borate, the activity was not evidently influenced by changing the TEA/TIBA ratio. In PP, Mt-I was about seven times more active than Mt-II, and produced PP with higher isotacticity than the latter. The M_w_ was evidently reduced by raising TEA content in the cocatalyst, meaning that chain transfer with the Al–Et bond should be faster than that with the Al–*i*Bu bond. Polyethylene MWD curve changed from mono-modal to bimodal type when TIBA was replaced by a different amount of TEA. PE produced by Mt-II/borate showed lower M_w_ and relatively narrow MWD. In PE, with Mt-II/borate/TIBA, vinyl ended PE accounted for about 70% of the chains, while in the Mt-I/borate/TEA system vinyl ended PE accounted for about 10%. It means that the Mt-I/borate/alkylaluminum system produced PE with dominantly saturated end groups regardless of the type of alkylaluminum. The data with kinetic modelling recommend that different types of active centers are present in PE system than PP. In PE, the [C*]/[Zr] of Mt-I/borate systems were higher than that of Mt-II/borate, but the Mt-II showed a higher ***k*_p_** value. Totally speaking, changing the TEA/TIBA ratio caused only limited influence on the kinetic parameters of Mt-I and Mt-II for PE. In PP, the Mt-I showed a much higher ([C*]/[Zr]) and *k_p_* value than the Mt-II. In Mt-I/borate, raising the TEA/TIBA ratio caused a reduction in *k*_p_, and the *k*_p_ of the Mt-I/borate/TEA system was less than 50% of the Mt-I/borate/TIBA system. 

## Figures and Tables

**Table 1:** Results of ethylene polymerization with Mt-I ^a^.

Run	Borate	Triethylaluminium (TEA)/Triisobutylaluminium (TIBA) (mol/mol)	Activity (kg/mmol_Mt_·h)	[C*]/[Zr] (%)	*k*_p_ (L/mol·s)	M_w_^b^ (kg/mol)	Ɖ ^b^	*T*_m_^c^ (°C)	Δ*H*_m_^c^ (J/g)
1.1	I	0/100	3.05	54.7	643	163	10.3	126	179
1.2	I	25/75	2.21	52.1	489	113	24.2	130	196
1.3	I	50/50	1.85	35.7	597	48	18.0	130	203
1.4	I	75/25	2.23	53.5	481	44	10.9	131	206
1.5	I	100/0	2.02	44.3	525	31	10.2	120	106
1.6	II	0/100	3.14	66.0	549	^n.d^	^n.d^	130	209
1.7	II	25/75	1.54	22.4	791	^n.d^	^n.d^	128	223
1.8	II	50/50	2.04	37.2	633	^n.d^	^n.d^	128	201
1.9	II	75/20	2.90	60.8	551	^n.d^	^n.d^	129	225
1.10	II	100/0	3.07	63.6	557	^n.d^	^n.d^	120	116

^a^ Reaction conditions: Mt-I = 1.25 μmol, Borate-I = 2.5 μmol, Borate-II = 2.5 μmol, ethylene pressure = 0.1 MPa, temperature = 50 °C, *t*_p_ = 20 min, solvent toluene = 50 mL, alkylaluminum (TEA, TIBA or TEA/TIBA mixture) 1000 μmol, 2-thiophenecarbonyl chloride (TPCC) = 2000 μmol, *t*_q_ = 5 min, ^b^ Determined by high temperature gel permeation chromatography (GPC), ^c^ Determined by differential scanning calorimetry (DSC), ^n.d^ Not determined.

**Table 2:** Results of ethylene polymerization with Mt-II ^a^.

Run	Borate	TEA/TIBA (mol/mol)	Activity (kg/mmol_Mt_·h)	[C*]/[Zr] (%)	*k*_p_ (L/mol·s)	*M*_w_^b^ (kg/mol)	Ɖ ^b^	*T*_m_^c^ (°C)	Δ*H*_m_^c^ (J/g)
2.1	I	25/75	3.22	44.3	837	2.4	2.2	122	214
2.2	I	25/75	3.22	44.3	837	2.4	2.2	122	214
2.3	I	50/50	3.02	51.4	678	8.2	3.3	106	54
2.4	I	75/25	3.72	54.7	785	2.6	2.2	123	212
2.5	I	100/0	3.41	50.1	785	2.9	2.5	101	156
2.6	II	0/100	3.00	43.6	793	^n.d^	^n.d^	122	226
2.7	II	25/75	2.90	47.1	712	^n.d^	^n.d^	122	170
2.8	II	50/50	5.02	57.5	1007	^n.d^	^n.d^	117	157
2.9	II	75/20	3.65	50.4	835	^n.d^	^n.d^	123	207
2.10	II	100/0	3.10	49.4	722	^n.d^	^n.d^	101	158

^a^ Reaction conditions: Mt-II = 1.25 μmol, Borate-I = 2.5 μmol, Borate-II = 2.5 μmol, ethylene pressure = 0.1 MPa, temperature = 50 °C, *t*_p_ = 20 min, solvent toluene = 50 mL, alkylaluminum (TEA, TIBA or TEA/TIBA mixture) 1000 μmol, TPCC = 2000 μmol, *t*_q_ = 5 min, ^b^ Determined by high temperature GPC, ^c^ Determined by DSC, ^n.d^Not determined.

**Table 3:** Results of propylene polymerization Mt-I/borate/alkylaluminum ^a^.

Run	Borate	TEA/TIBA (mol/mol)	Activity (kg/mmol_Mt_·h)	[C*]/[Zr] (%)	*k*_p_ (L/mol·s)	*M*_w_^b^ (kg/mol)	Ɖ ^b^	*T*_m_^c^ (°C)	Δ*H*_m_^c^ (J/g)
3.1	I	0/100	10.97	42.3	456	59.6	4.5	158	75
3.2	I	25/75	9.60	41.2	410	33.6	3.5	158	83
3.3	I	50/50	10.27	42.3	427	^n.d^	^n.d^	158	97
3.4	I	75/25	9.46	35.1	474	^n.d^	^n.d^	157	106
3.5	I	100/0	3.94	34.2	203	12.6	2.4	158	104
3.6	II	0/100	10.66	44.0	426	^n.d^	^n.d^	158	62
3.7	II	25/75	9.22	38.1	425	^n.d^	^n.d^	158	89
3.8	II	50/50	9.53	42.9	391	^n.d^	^n.d^	158	109
3.9	II	75/20	7.73	33.8	402	^n.d^	^n.d^	158	112
3.10	II	100/0	2.47	20.6	211	^n.d^	^n.d^	159	122

^a^ Reaction conditions: catalyst Mt-I = 1.25 μmol, Borate-I = 2.5 μmol, Borate-II = 2.5 μmol, propylene pressure = 0.1 MPa, temperature = 50 °C, *t*_p_ = 20 min, solvent toluene = 50 mL, coactivator: TEA, TIBA or TEA/TIBA mixture, Al = 1000 μmol, TPCC = 2000 μmol, *t*_q_ = 5 min, ^b^ Determined by high temperature GPC, ^c^ Determined by DSC, ^n.d^ Not determined.

**Table 4:** Results of propylene polymerization Mt-II/borate/alkylaluminum ^a^.

Run	Borate	TEA/TIBA (mol/mol)	Activity (kg/mmol_Mt_·h)	[C*]/[Zr] (%)	*k*_p_ (L/mol·s)	*M*_w_^b^ (kg/mol)	Ɖ ^b^	*T*_m_^c^ (°C)	*ΔH*_m_^c^ (J/g)
4.1	I	0/100	1.18	17.5	118	59.6	4.5	122	51
4.2	I	25/75	1.37	21.5	112	34.4	3.4	115	51
4.3	I	50/50	1.44	22.5	113	16.1	2.8	91	51
4.4	I	75/25	1.46	21.2	122	^n.d^	^n.d^	115	56
4.5	I	100/0	1.22	19.8	109	12.7	2.2	106	49
4.6	II	0/100	1.46	18.8	137	^n.d^	^n.d^	122	57
4.7	II	25/75	1.10	17.5	111	^n.d^	^n.d^	116	48
4.8	II	50/50	1.39	19.9	123	^n.d^	^n.d^	92	52
4.9	II	75/20	0.94	12.7	130	^n.d^	^n.d^	115	54
4.0	II	100/0	1.01	17.4	102	^n.d^	^n.d^	106	47

^a^ Reaction conditions: catalyst Mt-II = 1.25 μmol, Borate-I = 2.5 μmol, Borate-II = 2.5 μmol, propylene pressure = 0.1 MPa, temperature = 50 °C, *t*_p_ = 20 min, solvent toluene = 50 mL, coactivator: TEA, TIBA or TEA/TIBA mixture, Al = 1000 μmol, TPCC = 2000 μmol, *t*_q_ = 5 min, ^b^ Determined by high temperature GPC, ^c^ Determined by DSC, ^n.d^ Not determined.

**Table 5:** Comparisons between reported and recent experimental results under the same experimental conditions for Mt-II catalyzed polyethylene and polypropylene [26,30,39,59].

M	Cocatalyst	^a^ Activit	[C*]/[Zr] (%)	*k*_p_(L/mol·s)	*M*_w_^b^ (kg/mol)	Ɖ ^b^	*T*_m_^c^ (°C)	Δ*H*_m_^c^ (J/g)
E	Methylaluminoxane (MAO)	3.37	40.4	592	272	3.1	134.9	76.9
E	Methylaluminoxanes (MMAO)	2.16	67.8	330	195	3.0	^n.d^	^n.d^
E	dMAO	5.2	45.5	804	290	3.6	^n.d^	^n.d^
E	TIBA-borate-I	3.24	63.3	591	2.8	2.2	122	214
E	TEA borate-I	3.41	50.1	785	2.9	2.5	101	156
E	TIBA-borate-II	3.00	43.6	793	^n.d^	^n.d^	122	226
E	TEA borate-II	3.10	49.4	722	^n.d^	^n.d^	101	158
P	MMAO	3.8	24.3	156	15.2	1.8	^n.d^	^n.d^
P	TIBA-borate-I	1.18	17.5	118	59.6	4.5	122	51
p	TIBA borate-I	1.22	19.8	109	12.7	2.2	106	49
p	TEA-borate-II	1.46	18.8	137	n.d	n.d	122	57
p	TIBA borate-II	1.01	17.4	102	n.d	n.d	106	47

^a^ Activity (kg/mmol_Mt_·h), ^b^ Determined by high-temperature GPC, ^c^ Determined by DSC, ^n.d^ Not determined.

## Data Availability

Data will be made available on request.

## Supplementary Materials

The following are available online at https://www.mdpi.com/2073-4360/13/2/268/s1. Table S1 Number of PE chains formed by different chain transfer reactions a. Table S2 Distribution of Flory components in PE samples. Figure S1 Distribution of Flory components of PE produced from Mt-I/Borate-I with TEA/TIBA 0/100. Figure S2 Distribution of Flory components of PE produced from Mt-I/Borate-I with TEA/TIBA 25/75. Figure S3 Distribution of Flory components of PE produced from Mt-I/Borate-I with TEA/TIBA 50/50. Figure S4 Distribution of Flory components of PE produced from Mt-I/Borate-I with TEA/TIBA 75/25. Figure S5 Distribution of Flory components of PE produced from Mt-I/Borate-I with TEA/TIBA 100/0.
