# Supplementary material for: Kinetic and Thermal Study of Ethylene and Propylene Homo Polymerization Catalyzed by ansa-Zirconocene Activated with Alkylaluminum/Borate: Effects of Alkylaluminum on Polymerization Kinetics and Polymer Structure
Source: Polymers (Basel). 2021 Jan 15;13(2):268. doi: 10.3390/polym13020268 (PMC7830494; doi:10.3390/polym13020268)
Supplement: Supplementary file 1 [file polymers-13-00268-s001.pdf]

Kinetic and thermal study of ethylene and propylene homo polymerization catalyzed by *ansa*-zirconocene activated with alkylaluminum/borate: Effects of alkylaluminum on polymerization kinetics and polymer structure

Amjad Ali [1], Nadeem Muhammad [2], Shahid Hussain [1], Muhammad Imran Jamil [3], Azim Uddin [3], Tariq Aziz [3], Muhammad Khurram Tufail [2], Yintian Guo [3], Tiantian Wei [1], Ghulam Rasool [3], Zhiqiang Fan [3,*] and Li Guo [1,*]

[1] Research School of Polymeric Materials, School of Material Science & Engineering, Jiangsu University, Zhenjiang 202113, China; amjadali@zju.edu.cn (A.A.); shahid@ujs.edu.cn (S.H.); 2211905055@stmail.ujs.edu.cn (T.W.)
[2] Department of Enviromental Engineering, Wuhan University of Technology, Wuhan 430223, China; nadeem@zju.edu.cn (N.M.); Khurram.ch91@bit.edu.cn (M.K.T)
[3] MOE Key Laboratory of Macromolecular Synthesis and Functionalization, Department of Polymer Science and Engineering, Zhejiang University, Hangzhou 310027, China; jamil@zju.edu.cn (M.I.J.); auddin@zju.edu.cn (A.U.); Tariq_mehsud@yahoo.com (T.A.); .); ghulam46@yahoo.com (G.R.); guoyiantian@sinochem.com (Y.G.)
*Correspondence: fanzq@zju.edu.cn (Z.F.); liguo@ujs.edu.cn (L.G.)

Supporting information

Table S1 Number of PE chains formed by different chain transfer reactions[a].

run	Mt	AlR_3	N_{pol} (μmol)	N_v (μmol)	N_{Al} (μmol)	N_{Al}/N_{pol} (%)	M_n
1.1	I	TIBA	80	0	80	100	15960
1.3	I	TEA/TIBA (50/50)	290	0	290	100	2680
1.5	I	TEA	273	25	248	91	3080
2.1	II	TIBA	1409	1017	392	28	940
2.3	II	TEA/TIBA (50/50)	496	0	496	100	2540
2.5	II	TEA	1193	0	1193	100	1190

[a]-$N_v = N_{pol} \times I_v \times 2/(I_{CH2}/X_n)$, ($I_v$ is intensity of peaks at 4.8-5.1 ppm representing 2H of vinyl end group, I_{CH2} is intensity of peaks at 1.0-1.5 ppm representing 4H of an ethylene unit, $X_n = M_n/28$)

Table S2 Distribution of Flory components in PE samples.

Flory Component	TIBA		TEA/TIBA 25/75		TEA/TIBA 50/50		TEA/TIBA 75/25		TEA	
	M_w	F[a]	M_w	F[a]	M_w	F[a]	M_w	F[a]	M_w	F[a]
**A**			963.5	2						
**B**	560.3	13	436.8	9						
**C**	216.2	30	194.3	18	313.5	3	262.4	3		
**D**	84.1	26	92.7	18	127.1	15	109.6	14	112.6	9
**E**			45.3	13	57.9	26	49.9	29	50.5	29
**F**	30.4	17	16.7	10	26.4	15	24.1	22	23.1	21
**G**	9.4	12	6.8	13	8.5	10	8.7	17	8.2	18

**H**	2.7	3	2.4	14	2.4	17	2.7	7	2.6	11
**I**			1.0	5	1.0	15	0.8	8	0.9	13

[a] Weight fraction of Flory component, in unit of wt%.

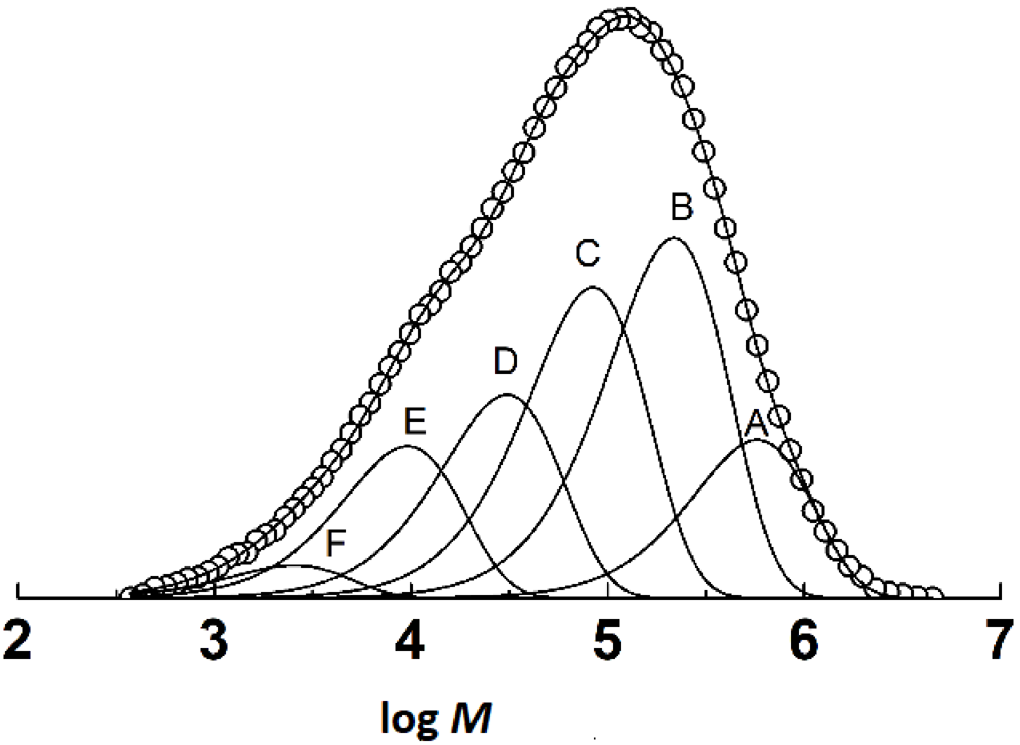

Figure S1 Distribution of Flory components of PE produced from Mt-I/Borate-I with TEA/TIBA 0/100.

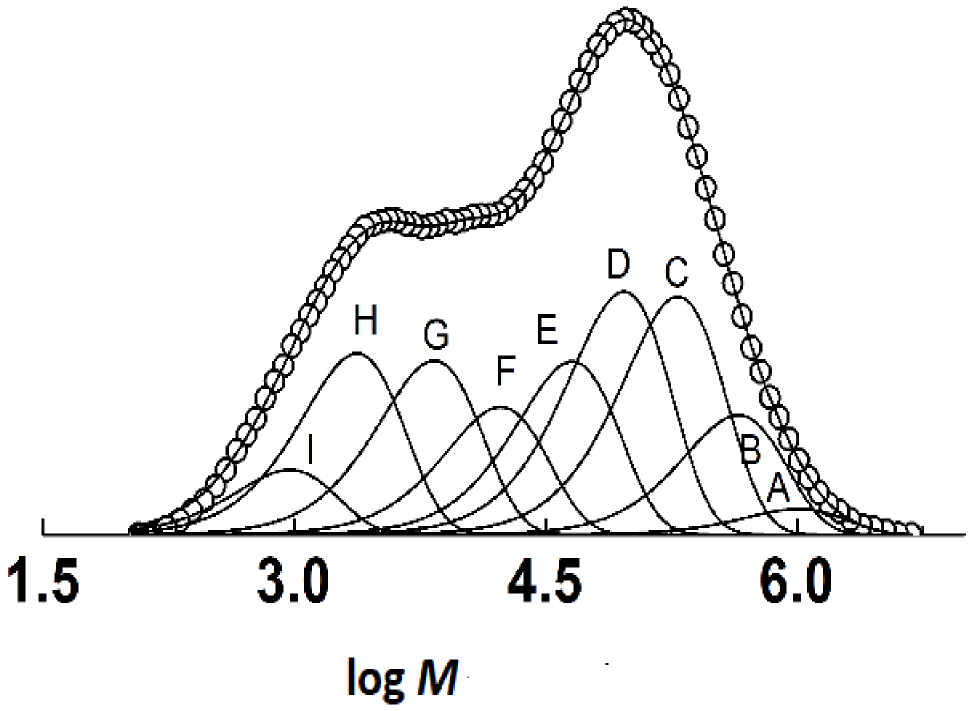

Figure S2 Distribution of Flory components of PE produced from Mt-I/Borate-I with TEA/TIBA 25/75.

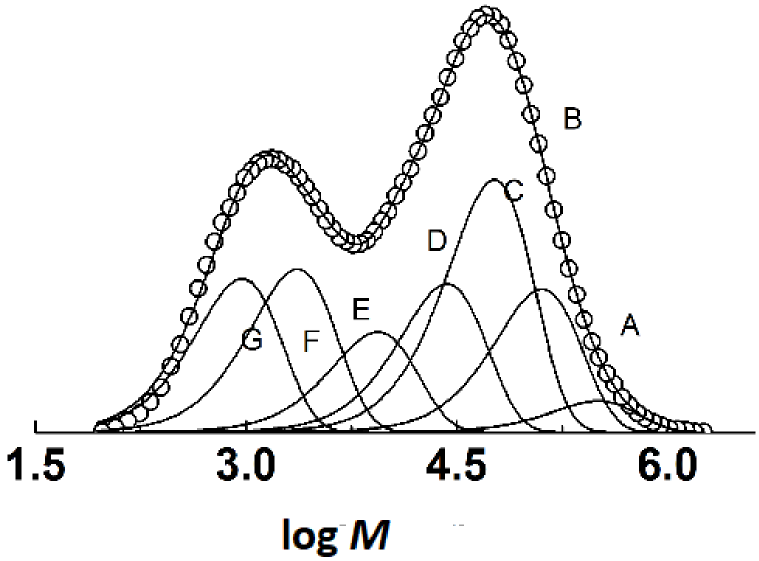

Figure S3 Distribution of Flory components of PE produced from Mt-I/Borate-I with TEA/TIBA 50/50.

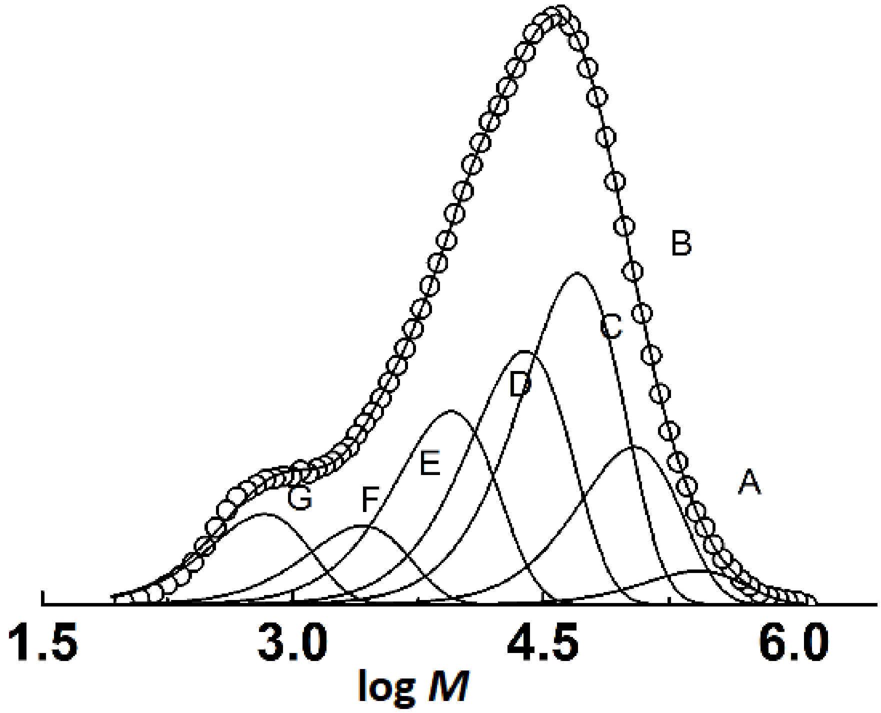

Figure S4 Distribution of Flory components of PE produced from Mt-I/Borate-I with TEA/TIBA 75/25.

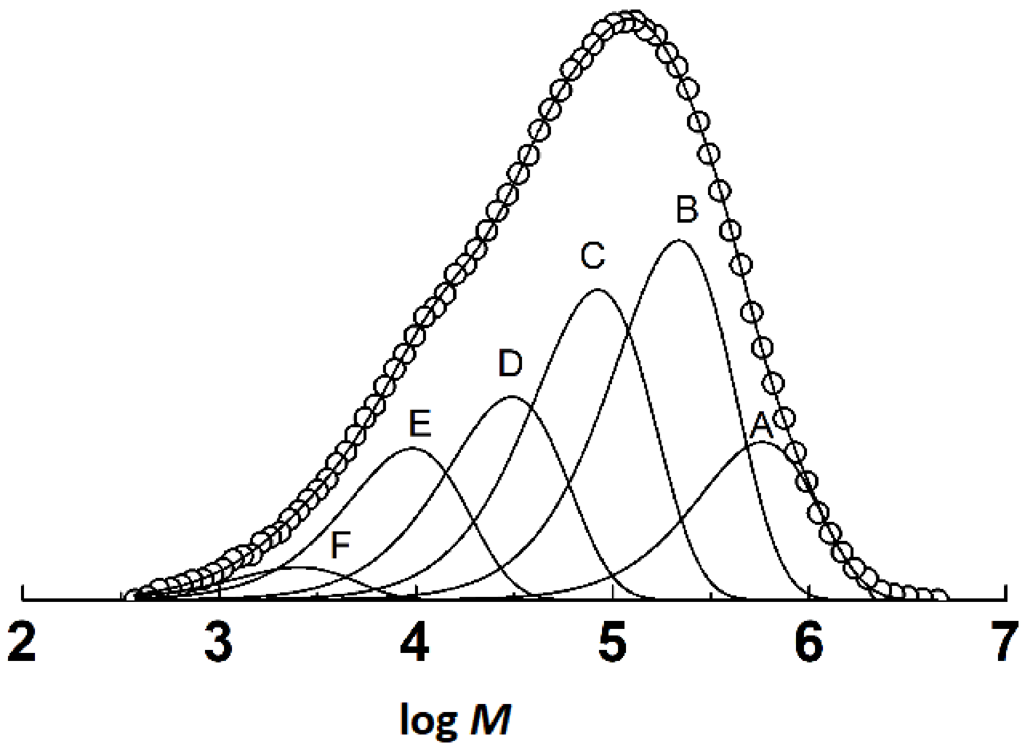

Figure S5 Distribution of Flory components of PE produced from Mt-I/Borate-I with TEA/TIBA 100/0.
